# Evaluation of the Performance of StypCel^TM^ Absorbable Hemostat for Intraoperative Hemorrhage Control in Neurosurgery: A Multicenter, Single-Arm Study in Riga, Latvia

**DOI:** 10.3390/medicina61101862

**Published:** 2025-10-16

**Authors:** Kaspars Auslands, Evelina Kocane, Evija Bergfelde, Egils Valeinis, Julija Dolgopolova, Jekabs Aksiks, Igors Aksiks

**Affiliations:** 1 Department of Neurology and Neurosurgery, Riga Stradins University, 1007 Riga, Latvia; 044632@rsu.edu.lv (E.K.); evija.bergfelde@aslimnica.lv (E.B.); 2 Department of Neurosurgery, Riga East Clinical University Hospital, 1038 Riga, Latvia; 3 Department of Neurosurgery, Pauls Stradins Clinical University Hospital, 1002 Riga, Latvia; egils.valeinis@stradini.lv (E.V.); julija.dolgopolova@stradini.lv (J.D.); 4 Department of Neurology and Neurosurgery, University of Latvia, 1586 Riga, Latvia; jekabsaksiks@gmail.com (J.A.); igors.aksiks@1slimnica.lv (I.A.); 5 Department of Neurosurgery, Riga’s 1st Hospital, 1001 Riga, Latvia

**Keywords:** StypCel™Absorbable Hemostat, bleeding control, plant-originated oxidized regenerated cellulose, non-woven, hemostatic gauze, neurosurgery

## Abstract

*Background and Objectives:* Intraoperative bleeding during neurosurgical procedures poses a significant risk by increasing morbidity and mortality, obscuring the surgical field and prolonging operative time and hospitalization. Effective hemostasis is therefore essential, frequently necessitating the use of topical hemostatic agents. This study aimed to evaluate the performance of a plant-derived oxidized regenerated cellulose (ORC) hemostatic agent StypCel™ Absorbable Hemostat (Medprin Regenerative Medical Technologies Co., Ltd.) in various neurosurgical interventions, including intracranial tumor resections, spinal surgeries, trigeminal neuralgia operations, cerebrospinal fluid fistula repair and ventriculoperitoneal shunt implantation. The study aimed to assess its performance in these procedures due to the high risk of intraoperative bleeding and the challenges of achieving hemostasis in delicate neural structures. *Materials and Methods:* This prospective, single-arm clinical study included 46 patients who underwent neurosurgical procedures at three neurosurgerical clinics in Riga, Latvia. The primary endpoint was the rate of effective bleeding control achieved within 5 min of StypCel™ application. Safety assessments included monitoring for central nervous system infections (CNSI), intracranial granuloma formation, new-onset neurological deficits, seizures, anaphylactic reactions or device malfunction. All adverse events (AEs) and serious adverse events (SAEs) were documented during the postoperative follow-up. *Results:* The cohort consisted of 46 patients (29 females and 17 males), including 20 with neoplastic intracranial lesions and 26 with other neurosurgical pathologies. Effective bleeding control within 5 min was achieved in 93.5% of cases (95% CI: 82.1–98.6%). In three patients, bleeding control exceeded 5 min due to unexpected arterial hemorrhage encountered during intracranial tumor resection. No device-related AEs, SAEs, CNSIs or granuloma formations were reported throughout the follow-up period. *Conclusions:* The findings demonstrate that StypCel™ Absorbable Hemostat is a safe and effective adjunct for achieving intraoperative hemostasis in neurosurgical procedures. Its favorable safety profile and high hemostatic success rate support its clinical utility, particularly for controlling low-pressure venous or capillary bleeding. Further comparative and long-term studies are warranted to validate these results in broader surgical settings.

## 1. Introduction

Intraoperative hemorrhage is a common and potentially life-threatening complication encountered across a range of neurosurgical procedures. The estimated incidence rate is between 10% and 30% depending on the type of procedure and individual patient factors [1]. Uncontrolled hemorrhage not only impairs the surgeon’s visual field but also contributes to prolonged operative duration, increased risk of postoperative neurological deficits and elevated perioperative morbidity and mortality [2]. While electrocautery and mechanical ligation are standard techniques to achieving hemostasis, their effectiveness may be limited due to restricted anatomical access, poor visibility or the proximity of critical neurovascular structures, thereby necessitating adjunctive hemostatic strategies.

Systemic hemostatic agents, including antifibrinolytic medications and coagulation factor concentrates, may offer effective control in cases of diffuse bleeding. However, their use is associated with significant risks, such as thrombembolic events, hypersensitivity reactions and alterations in systemic hemostatic balance. Similarly, the administration of blood components is reserved for specific clinical indications and is not without risk, being associated with immunologic complications, volume overload and the potential transmission of infectious agents [3,4].

Anticoagulation management in neurosurgical patients is an increasingly relevant clinical challenge. For example, in patients with high-grade gliomas the thrombotic risk is elevated but anticoagulant therapy carries an increased risk of intralesional bleeding. A systematic review by Bianconi et al. (2021) reported that in patients receiving anticoagulation for thromboembolic events, the risk of intracranial hemorrhage ranged from 0% to 15.4% while the risk of venous thromboembolism (VTE) ranged between 4% and 33% [5]. These findings underscore the need to carefully balance hemorrhagic and thrombotic risks in neurosurgical procedures, highlighting the importance of effective intraoperative hemostatic agents.

In contrast, topical hemostatic agents offer the advantage of localized action at the bleeding site, reducing the likelihood of systemic side effects while facilitating rapid intraoperative bleeding control in delicate surgical environments such as the central nervous system [6,7]. A variety of topical hemostatic agents have been developed to address the specific and evolving needs of modern surgical practice. Their effectiveness varies considerably depending on the material composition, structural form and underlying mechanism of action. Among these, oxidized regenerated cellulose (ORC) has emerged as a widely used plant-derived biomaterial [8]. ORC functions by providing a physical scaffold that enhances platelet aggregation and fibrin deposition at the bleeding site, ultimately facilitating clot formation [9]. StypCel^TM^ was invisible to the naked eye at 1 month.

StypCel™ Absorbable Hemostat is a soft, non-woven, multilayered fabric composed of plant-derived oxidized regenerated cellulose (ORC). It is applied in its dry form directly to the bleeding surface where it conforms to irregular anatomical structures. Unlike traditional knitted ORC products, the non-woven structure of StypCel^TM^ allows for improved adaptability and precision, allowing surgeons to customize the material’s size and shape to conform to irregular or anatomically constrained bleeding sites. Once hemostasis is achieved, any excess material is carefully removed to promote complete absorption and minimize foreign body reaction.

The primary aim of this study is to evaluate the clinical performance of StypCel^TM^ as a topical hemostatic agent in neurosurgical procedures in three neurosurgery clinics in Latvia–Riga East Clinical University Hospital, Pauls Stradins Clinical University Hospital and Riga’s 1st Hospital.

## 2. Materials and Methods

This prospective, single-arm clinical study included 46 patients who underwent neurosurgical procedures at three neurosurgery clinics in Riga, Latvia, as a part of multicenter international post-market clinical follow-up investigation. The participants were recruited from the Departments of Neurosurgery at three institutions in Latvia–Riga East Clinical University Hospital (RECUH), Pauls Stradins Clinical University Hospital (PSCUH) and Riga’s 1st Hospital (R1H). The enrollment period extended from 7 June 2023 to 26 September 2024.

Eligible participants were adults aged 18 to 75 years who required a topical hemostatic agent during a neurosurgical intervention. Patients were excluded if they presented with underlying osseus defects, hemorrhage from major arterial vessels or if use of StypCel^TM^ was contraindicated (for example, intended use as an anti-adhesion barrier, application on non-hemorrhagic serous surfaces). Additionally, patients requiring non-removable implants or packing materials were excluded from the study.

Patient data were extracted from physical medical records and the electronic clinical database system “Ārsta Birojs” at RECUH and PSCUH. Collected data included sociodemographic characteristics, procedural details and relevant clinical parameters.

Surgical interventions were performed in operating rooms under standard aseptic conditions, in accordance with established institutional protocols and neurosurgical best practices. The use of StypCel^TM^ was determined at the discretion of the operating neurosurgeon when conventional hemostatic methods (such as suturing, vessel ligation or electrocautery) were deemed inadequate, impractical or potentially hazardous due to anatomical constraints or proximity to critical neurovascular structures.

The application technique, including the surface area covered and the number of layers used, was tailored to the severity of intraoperative bleeding with the principle of utilizing the minimal effective amount to achieve hemostasis. StypCel^TM^ was recommended to be applied in its dry form. Once hemostasis was achieved, any excess material was removed from the surgical field prior to wound closure in order to promote complete absorption and reduce the risk of foreign body response.

The primary endpoint, defined as the achievement of hemostasis within 5 min after StypCel™ application, was assessed by direct visual inspection. Hemostasis was considered successful if no active bleeding was observed at the treated site at any time within the 5 min observation period. Gentle tamponade with a sterile swab for up to 1 min could be applied if bleeding continued immediately after StypCel™ application but the maximum observation period to determine success was 5 min.

StypCel^TM^ Absorbable Hemostat (Medprin Regenerative Medical Technologies Co., Ltd., Guangzhou, China) is a bioabsorbable topical hemostatic agent composed of oxidized regenerated cellulose (ORC) presented in a non-woven, multilayered fabric form. The material is soft, pliable and ranges in color from white to slightly yellow, allowing for precise adaptation to irregular surgical fields. Its hemostatic effect is primarily mechanical and physicochemical, promoting clot formation by transforming into a gelatinous matrix upon contact with blood, thereby facilitating platelet adhesion and aggregation. StypCel^TM^ is indicated for adjunctive use in controlling capillary, venous and small arterial bleeding where conventional techniques are ineffective or impractical. The product undergoes enzymatic degradation into glucose and glucuronic acid which are subsequently metabolized and absorbed by the body without significant inflammatory response.

To assess the clinical performance of StypCel™ Absorbable Hemostat in neurosurgery, the hemostatic agent was applied in the most frequently performed neurosurgical procedures in the Latvian patient population, including spinal surgeries (such as microdiscectomy and over-the-top spinal canal decompression) and intracranial tumor resections. In addition, its applicability was evaluated in less commonly performed procedures, including surgeries for trigeminal neuralgia, ventriculoperitoneal shunt implantation and the closure of cerebrospinal fluid (CSF) fistulas. This approach allowed us to capture both routine and specialized neurosurgical interventions, providing a broader perspective on the agent’s performance across a heterogeneous range of procedures.

The study was conducted in accordance with the CONSORT/TREND guidelines for clinical research reporting. The clinical trial is registered at ClinicalTrials.gov under the identifier NCT07199439 (first posted on 30 September 2025).

All statistical analyses were performed using SAS Software version 9.4. Continuous variables were summarized using descriptive statistics, including mean, standard deviation (SD), median, interquartile range (IQR), minimum and maximum values. Categorical variables were expressed as frequencies and percentages, with percentages calculated based on available (non-missing) data. The primary endpoint was analyzed using exact binomial 95% confidence intervals or CI (Clopper–Pearson method). Accordingly, formal hypothesis testing and *p*-values were not performed, instead the results are presented descriptively with confidence intervals included to indicate the precision of the estimates. For continuous variables, confidence intervals were derived using the t-distribution while the Clopper–Pearson method was also applied to categorical outcomes. No formal hypothesis testing, *p*-values or subgroup comparisons were performed. Results are presented descriptively with confidence intervals to indicate the precision of the estimates.

## 3. Results

### 3.1. Demographics Characteristics

A total of 46 patients were enrolled in the study. The median age of the cohort was 55.8 years (Q1–Q3 48.0–69.0). Regarding gender distribution, 63.0% (n = 29) of the participants were female, while 37.0% (n = 17) were male. The majority of patients were identified as Caucasian (71.7%, n = 33).

All patients underwent neurosurgical procedures during which StypCel^TM^ was applied as a topical hemostatic agent. StypCel^TM^ was most frequently utilized during intracranial tumor resections (43.5%, n = 20) and neurosurgical interventions addressing spinal disorders (41.3%, n = 19). It was also applied in surgeries for trigeminal neuralgia (8.7%, n = 4), ventriculoperitoneal shunt implantantion (4.3%, n = 2) and in the surgical closure of a cerebrospinal fluid (CSF) fistula (2.2%, n = 1).

Comorbidities were documented in 27 patients (58.7%). The most commonly reported categories included vascular disorders (39.1%, n = 18), followed by metabolism and nutrition disorders (13.0%, n = 6), cardiac disorders (10.9%, n = 5) and conditions related to surgical and medical procedures (10.9%, n = 5). Other reported comorbidities included nervous system disorders (8.7%, n = 4), endocrine disorders (4.3%, n = 2), respiratory, thoracic and mediastinal disorders (4.3%, n = 2), benign and malignant neoplasms (4.3%, n = 2), musculoskeletal and connective tissue disorders (4.3%, n = 2), general disorders and administration site conditions (2.2%, n = 1), infectious diseases (2.2%, n = 1) and injuries (2.2%, n = 1). It is important to note that vascular disorders were observed in 18 patients within the cohort including primary arterial hypertension (n = 16), atherosclerosis (n = 4) and lymphostasis (n = 1). All demographics characteristics, causes of surgery and comorbidities are summarized in Table 1.

A number of study participants were receiving pharmacological treatment prior to surgery, some of which was discontinued before the neurosurgical intervention; such medications included gabapentin, doxycycline and ipidacrine.

In total, 56.5% (n = 26) of patients continued pharmacotherapy concomitantly with the surgical procedure. The most commonly used therapeutic classes were beta blocking agents (26.1%, n = 12), lipid modifying agents (17.4%, n = 8), agents acting on the renin-angiotensin system (15.2%, n = 7), anti-inflammatory and antirheumatic agents (13.0%, n = 6) and diuretics (10,9%, n = 5). Other concomitantly used medications included antidiabetic drugs (8.7%, n = 4), antithrombotic agents (6.5%, n = 3), calcium channel blockers (6.5%, n = 3), analgesics, anti-parkinson drugs, antibacterial for systemic use, antiepileptics, antihypertensives, thyroid therapy and drugs for acid-related disorders (each 4.3%, n = 2). Less frequently reported were cardiac therapy, corticosteroids for systemic use, digestive enzymes and drugs for obstructive airway diseases (each 2.2%, n = 1).

### 3.2. Hemostasis Results

The duration of hemostasis was measured from the moment the entire bleeding surface was fully covered with the hemostatic material until the point when no active bleeding (errhysis) was observed. Hemostasis was defined as the complete cessation of bleeding indicated by the absence of fresh blood spreading from the application site or the presence of dark coagulated spots without further expansion.

Hemostasis was achieved within 5 min in the majority of cases, resulting in a primary performance rate of 93.5% (95% CI: 82.1–98.6%). In three cases, the time to bleeding control exceeded 5 min during complex intracranial tumor resections where intraoperative hemorrhage was more extensive than anticipated. The mean time required to achieve effective hemostasis across all patients was 133.5 ± 92.93 s, while the median time was 121.0 s with the fastest response observed at just 23 s. These findings are summarized in Table 2. These results are presented descriptively, without formal hypothesis testing or subgroup comparisons, and indicate that StypCel™ provided rapid and consistent bleeding control under typical neurosurgical conditions.

Body temperature was recorded at two perioperative time points—upon patient arrival in the operating room and during postoperative observation in the recovery room. Normothermia (36.0–37.0 °C) was maintained in all patients at both time points. To support temperature maintenance warming blankets set to 38.0–40.0 °C were used and the operating room ambient temperature was maintained at 22.0–23.0 °C. These measures were sufficient to preserve normothermia throughout the surgical procedure.

An assessment of hemostasis effectiveness was also conducted, involving the initial 20 patients enrolled in the roll-in set group. Surgeons evaluated their satisfaction with the StypCel^TM^ hemostatic performance using a five-point Likert scale “Very satisfied”, “Somewhat satisfied”, “Neutral”, “Somewhat dissatisfied” and “Very dissatisfied”. The majority of cases (65.0%, n = 13) were rated as “Very satisfied” while the remaining 35.0% (n = 7) were assessed as “Somewhat satisfied”. Notably, none of the cases received a “Neutral”, “Somewhat dissatisfied” and “Very dissatisfied” rating.

Overall StypCel^TM^ demonstrated a favorable safety profile throughout the postoperative course. During two follow-up assessments (on postoperative day 10 and at 6 months), no signs of intracranial infection or delayed complications were observed. Furthermore, no causes of granuloma formation, new-onset neurological deficits, seizures, anaphylactic reactions or device malfunction were reported. Importantly, no patients experienced device-related adverse events (AEs) or serious adverse events (SAEs) and no deaths occurred during the study period. All patients achieved satisfactory postoperative recovery, supporting the overall clinical safety and tolerability of StypCel in neurosurgical applications.

## 4. Discussion

This prospective, single-arm post-market clinical study evaluated the performance of StypCel^TM^ Absorbable Hemostat in the different neurosurgical interventions. The findings demonstrated favorable performance with a hemostatic success rate of 93.5% achieved within 5 min of application. The mean time to hemostasis was 133.5 ± 92.93 s, indicating rapid bleeding control. No adverse events beyond those typically associated with standard postoperative neurosurgical recovery were reported. Importantly, no cases of central nervous system infection, intracranial granuloma formation or serious device-related adverse events were observed throughout the study period, supporting the safety and clinical utility of StypCel^TM^ in neurosurgical practice.

Previous preclinical studies and early-stage clinical trials of StypCel™ Absorbable Hemostat demonstrated its ability to rapidly achieve hemostasis in low-pressure bleeding models with minimal local tissue reaction. It should be noted that the information regarding these preclinical and early-stage studies is based on data provided by the manufacturer as peer-reviewed publications on these studies are limited or not publicly available.

In numerous studies, it has been consistently highlighted that the use of oxidized regenerated cellulose (ORC) across various surgical specialties may be associated with both early and delayed complications, including allergic reactions, seroma formation, foreign body responses leading to compressive neuropathies and misdiagnose during follow-up [10]. While these hemostatic agents are typically safe and well-tolerated, they should be removed when applied near foramina, confined bony spaces, the spinal cord or the optic nerve and chiasm, as they may swell post-application and exert undesirable pressure on adjacent structures [11,12,13]. Nevertheless, in certain cases surgeons may elect to leave a minimal amount of the hemostatic agent in the surgical field. Menovsky et al. (2011) reported a clinical case of mass effect following the use of Surgicel Fibrillar in spinal surgery, where postoperative massive swelling of the hemostatic material exerted compression on the dura mater [11]. Additional case reports have described instances of postoperative paraplegia [14,15,16] and radiculopathy [12] attributed to intraoperative ORC use.

In our study, the primary data collected were limited to sociodemographic characteristics, time to achieve hemostasis and the incidence of postoperative complications, representing a relatively narrow scope of investigation. In contrast, Qian et al. (2020) conducted a comparative study evaluating two patients cohorts—one in which oxidized regenerated cellulose (ORC) was applied intraoperatively as a topical hemostatic agent and another in which no such adjunctive material was used [17]. Their analysis encompassed multiple surgical specialties, including neurosurgery, where they specifically examined subgroups undergoing endoscopic transnasal transsphenoidal surgery (ESTSS), non-skull base craniotomy (NSBC) and cerebrovascular surgery (CVS). The results demonstrated that patients in the non-ORC groups required higher blood transfusion (by 20.0% in ESTSS and 19.0% in NSBC) and incurred higher hospital costs (by 7.4% in ESTSS and 13.2% in NSBC) compared to the ORC groups. A statistically significant difference was observed in hospital costs for NSBC procedures (*p* = 0.002). Interestingly, for CVS the non-ORC group showed a 26.4% lower frequency and 19.8% lower volume of blood transfusion than the ORC group. However, CVS the non-ORC group hospitalization costs were 8.7% higher than in the ORC group (*p* = 0.156) [16]. Such comparative analyses, especially those evaluating resource consumption and total healthcare costs, emphasize the potential clinical and economic benefits of using ORC. Including similar comparisons and cost-effectiveness assessments in future research could offer a more thorough understanding of the utility of ORC in neurosurgical procedures.

It is well established that vascular pathologies, due to mechanisms such as intravascular coagulation or an increased risk of venous thrombosis, predispose patients to intraoperative bleeding complications. In this cohort, vascular comorbidities were identified in 18 patients, specifically primary arterial hypertension (n = 16), atherosclerosis (n = 4) and lymphostasis (n = 1). Notably, only three of these patients were receiving antithrombotic therapy. One patient had a history of myocardial infarction in 2015 and currently suffered from arterial hypertension and atherosclerosis. He had been prescribed acetylsalicylic acid (*Aspirin*) 100 mg orally once daily but discontinued its use seven days prior to surgery. Another patient presented with chronic heart failure, arterial hypertension, coronary artery disease, atherosclerosis and atrial fibrillation, he was on direct oral anticoagulant (DOAC) therapy with edoxaban (*Lixiana*) 20 mg orally once daily, which was stopped two days before the surgery. The third patient, with lymphostasis of the right leg, had been treated with rivaroxaban (*Xarelto*) 20 mg orally once daily which was also discontinued two days before the surgery. In this study, the success rate of hemostasis in these particular patients was not analyzed in detail. However, it is important to emphasize that even after discontinuation of anticoagulant or antiplatelet therapy patients may remain at risk of both increased perioperative bleeding and hypercoagulability.

It is well recognized that coagulation function is closely influenced by body temperature, which is particularly critical in the perioperative period. In this study, body temperature was measured at the beginning of the perioperative period upon patient arrival in the operating room and at the end of the perioperative period during postoperative observation in the recovery room. In all patients, normothermia (36.0–37.0 °C) was observed at both time points. To support maintenance of normothermia, warming blankets with a temperature range of 38.0–40.0 °C were applied and the operating room ambient temperature was set to 22.0–23.0 °C. Under standard conditions, these measures are generally sufficient to preserve normothermia throughout the surgical procedure.

Emerging hemostatic materials could be evaluated not only through clinical endpoints but also by investigating their detailed cellular effects on biological tissues. Kleine et al. (2021) emphasized that novel ORC-based materials such as Tabotamp, Equicel and Equitamp may be studied based on their physicochemical characteristics, including structural morphology, solubility, pH levels and specific effects on neuronal tissue [18]. Cytotoxicity was assessed using DNA-binding fluorescent dyes in Schwann cells, astrocytes and neurons. Additionally, organotypic hippocampal slice cultures (OHSCs) were employed, utilizing propidium iodide, hematoxylin-eosin and isolectin B4 staining to evaluate tissue damage, cytoarchitecture and microglial activation. Overall, the findings support the tailored use of individual ORC formulations in specific clinical contexts based on their distinct properties. However, further clinical investigations are warranted to confirm and compare the complication profiles of different ORC products, particularly when applied in eloquent or functionally critical regions of the central nervous system [18].

Numerous studies have compared various members of the Surgicel product family (such as Surgicel Original, Surgicel Fibrillar, Surgicel Nu-Knit, Surgicel SNoW and Surgicel Powder) highlighting their roles as adjuncts to standard hemostatic techniques [13,19]. These oxidized regenerated cellulose (ORC)-based agents not only promote rapid hemostasis but also exhibit bactericidal activity, including efficacy against antibiotic-resistant pathogens such as methicillin-resistant Staphylococcus aureus (MRSA), Staphylococcus epidermidis, Streptococcus pneumoniae and Pseudomonas aeruginosa [19]. Although our study did not include a direct head-to-head clinical comparison, previous in vivo preclinical studies with Surgicel (Stark et al., 2024) demonstrated that hemostasis within 5 min was achieved in approximately 85–88% of bleeding sites in liver and spleen defect models [20]. In our postmarket study, StypCel™ Absorbable Hemostat achieved hemostasis within 5 min in 93.5% of cases, indicating rapid and reliable bleeding control. These results suggest that StypCel™ provides comparable or slightly superior hemostatic success rate to Surgicel in standardized models and supports its use as a safe and effective adjunct for managing intraoperative bleeding in neurosurgical procedures.

In our study we evaluated the rate of effective bleeding control achieved within 5 min. In contrast, Al-Attar et al. (2023) [13] investigated the safety and hemostatic performance of Surgicel Powder by assessing outcomes at 3, 5 and 10 min after applying. Their reported success rates were 77.7%, 84.7% and 92.2%, respectively, providing a more dynamic overview of the progression of hemostasis and the risk of rebleeding following initial hemostatic control. Additionally, the study incorporated a surgeon ease-of-use questionnaire completed within 72 h after surgery. This seven-question survey evaluated the usability of Surgicel Powder at the target bleeding site (TBS) using a Likert-type scale ranging from “Disagree” to “Strongly agree”. Such instruments are valuable for capturing surgeon experience and subjective practicality which are essential considerations when assessing the clinical integration and ergonomic utility of novel hemostatic agents [13]. The Likert scale evaluation was limited to the roll-in group of the first 20 patients to gain initial practical experience with the use of StypCel™ and to capture early feedback on surgeon satisfaction and usability. By focusing on this smaller initial cohort, we were able to monitor and refine the application technique and assess user perceptions in a controlled manner before applying the scale to the broader study population. This approach is consistent with standard practice in postmarket observational studies where early user feedback is collected during an initial roll-in phase.

Additionally, the use of a standardized bleeding severity scale, such as the Bleeding Academic Research Consortium (BARC) scale, could enhance the evaluation of hemorrhagic outcomes in clinical trials. The BARC scale categorizes bleeding into five grades ranging from mild, asymptomatic bleeding that does not require intervention to severe or life-threatening hemorrhage necessitating urgent medical management, massive blood transfusions or resulting in death. Incorporating this scale into our study would allow for a more objective assessment of blood loss during neurosurgical procedures in which StypCel was applied, thereby improving the rigor and comparability of outcome measures.

In this study, StypCel™ Absorbable Hemostat was applied in the most commonly performed neurosurgical procedures in the Latvian patient population, including spinal surgeries (such as microdiscectomy and over-the-top spinal canal decompression) and intracranial tumor resections. Its use was also evaluated in less frequently performed procedures, including surgeries for trigeminal neuralgia, ventriculoperitoneal shunt implantation and cerebrospinal fluid (CSF) fistula closure. This approach provided insight into the agent’s performance across both routine and specialized neurosurgical interventions, reflecting a heterogeneous range of clinical scenarios. For future studies, a more detailed subgroup analysis could be considered. In spinal surgeries, the specific spinal segment involved (cervical, thoracic, lumbar or sacral), the surgical approach used (anterior, lateral or posterior) and the number of operated levels (single-level or multilevel) could be examined. For intracranial tumor resections, the tumor location (anterior, middle or posterior cranial fossa) and whether an endoscopic approach was used could be further analyzed. Additionally, in all types of neurosurgical procedures it would be valuable to distinguish between primary and repeat operations, as reoperations often involve scar tissue that may increase the risk of intraoperative bleeding. Such detailed analyses could provide a more nuanced understanding of StypCel™ performance in specific procedural contexts.

Notably, in three cases within the current study, the time to achieve hemostasis exceeded five minutes, primarily due to unforeseen arterial bleeding encountered during intracranial tumor resection. These cases underscore the limited performance of oxidized regenerated cellulose (ORC) based hemostats such as StypCel^TM^ in managing high-flow arterial hemorrhage, consistent with previous findings that suggest ORC products are more effective in low-pressure capillary or venous bleeding rather than in arterial sources.

From a clinical perspective, StypCel™ offers several potential benefits: its soft, non-woven, multilayered structure allows precise adaptation to irregular or anatomically constrained bleeding sites minimizing trauma to surrounding neural tissue. It can be applied in dry form without preparation, facilitating rapid intraoperative deployment and reducing operative time. From a logistical perspective, the ability to easily tailor the size and shape of the hemostatic material, combined with its predictable absorption and minimal foreign body reaction may improve surgical workflow and efficiency in neurosurgical procedures.

Our study has several limitations that should be considered. First, it employed a single-arm design without a control or comparator group, preventing direct comparison of StypCel^TM^ performance with other hemostatic agents. Second, the observational and non-comperative nature of the results means that the findings primarily describe practical use rather than provide statistical evidence of comparative efficacy or superiority. Third, the measurement of the primary endpoint (achievement of hemostasis within 5 min) may be subject to observer variability and potential subjectivity. Fourth, the cohort was limited to 46 patients which may be insufficient to detect rare or very rare complications associated with StypCel^TM^ use. Fifth, the follow-up period was relatively short and the follow-up protocol was not standardized across all patients, limiting the ability to assess long-term outcomes and detect delayed adverse events, such as granuloma formation, which can take years to develop. Sixth, the study did not include an evaluation of cost-effectiveness, such as hospital costs or overall resource utilization which may be important for broader clinical and economic considerations. Finally, it should be noted that there is a limited body of peer-reviewed, scientifically validated literature on the use of StypCel™ in neurosurgery and other surgical specialties and much of the currently available information is derived from data provided by the manufacturer. These issues highlight key aspects that could be improved in subsequent research.

## 5. Conclusions

StypCel^TM^ demonstrated a high rate of hemostatic performance, achieving effective bleeding control within clinically acceptable timeframes. The safety profile was favorable, with no significant adverse events or complications observed throughout the 6-month postoperative follow-up. These findings support the effectiveness and safety of StypCel^TM^ Absorbable Hemostat as an adjunctive hemostatic agent for managing intraoperative bleeding in neurosurgical interventions.

## Figures and Tables

**Table 1:** Demographics characteristics, causes of surgery and comorbidities.

	n (%)
**Age in years (mean)**	55.8 ± 13.65
**Gender**	
Female	29 (63.0%)
Male	17 (37.0%)
**Race**	
Caucasian	33 (71.7%)
Other	13 (28.3%)
**Causes of surgery**	
Intracranial tumor	20 (43.5%)
Spinal disorder	19 (41.3%)
Trigeminal neuralgia	4 (8.7%)
Ventriculoperitoneal shunt implantation	2 (4.3%)
Surgical closure of a cerebrospinal fluid (CSF) fistula	1 (2.2%)
**Comorbidities**	
Vascular disorders	18 (39.1%)
Metabolism and nutrition disorders	6 (13.0%)
Cardiac disorders	5 (10.9%)
Conditions related to surgical and medical procedures	5 (10.9%)
Nervous system disorders	4 (8.7%)
Endocrine disorders	2 (4.3%)
Respiratory, thoracic and mediastinal disorders	2 (4.3%)
Benign and malignant neoplasms	2 (4.3%)
Musculoskeletal and connective tissue disorders	2 (4.3%)
General disorders and administration site conditions	1 (2.2%)
Infectious diseases	1 (2.2%)
Injuries	1 (2.2%)

**Table 2:** Hemostasis success rate and time.

**Hemostasis success rate**	
n/N (%)	43/46 (93.5%)
95% CI ^1^	82.1–98.6%
**Time to hemostasis (s)**	
Mean ± SD	133.5 ± 92.93
Median	121.0
Minimum value	23.0
Maximum value	425.0
Q1–Q3 ^2^	65.0–156.0

^1^ CI: Clopper-Pearson method. ^2^ Q1: first quartile; Q3: third quartile.

## Data Availability

Data are available upon request due to ethical restrictions. All the data included in this study are available upon request from the corresponding author. The data are not publicly available and are stored in the patient medical record repository at Riga East Clinical University Hospital, Pauls Stradins Clinical University Hospital and Riga’s 1st Hospital according to where the individual patient was treated.

## Abbreviations

The following abbreviations are used in this manuscript:

VTE	Venous thromboembolism
ORC	Oxidized regenerated cellulose
RECUH	Riga East Clinical University Hospital
PSCUH	Pauls Stradins Clinical University Hospital
R1H	Riga’s 1st Hospital
SD	Standard deviation
IQR	Interquartile range
CI	Clopper-Pearson method
AEs	Adverse events
SAEs	Serious adverse events
ESTSS	Endoscopic transnasal transsphenoidal surgery
NSBC	Non-skull base craniotomy
CVS	Cerebrovascular surgery
TBS	Target bleeding site
